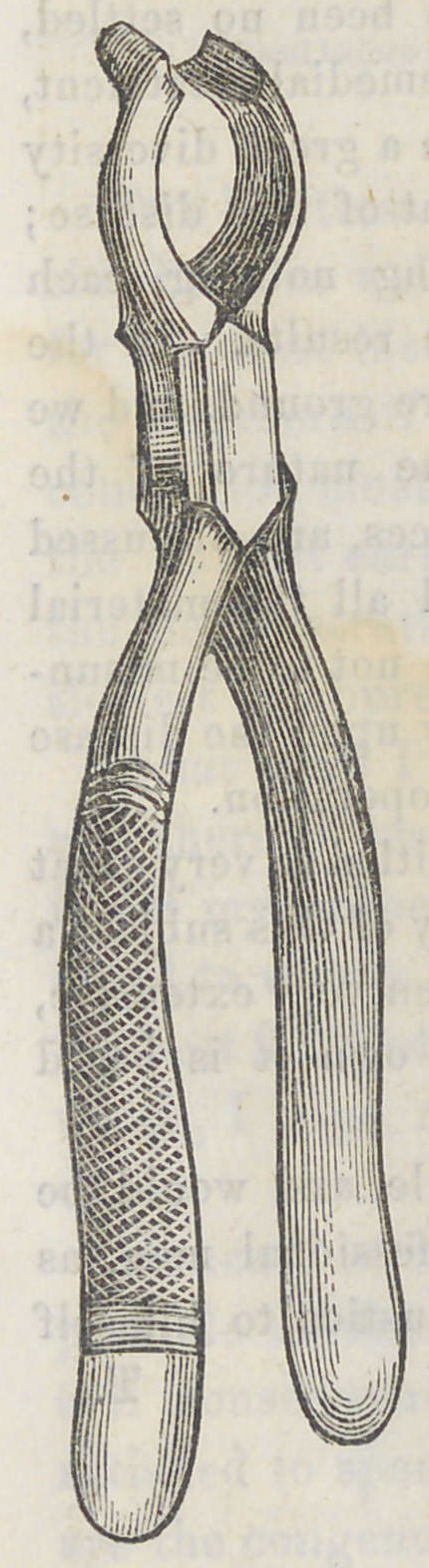# Editorial

**Published:** 1861-04

**Authors:** 


### Editorial.

### DENTAL PATENTS.

Our correspondent in the March number of the Register, takes exceptions to our remarks on Dental Patents in the December number, or at least he says he can not agree with the tenor of our article. But we think he has hardly touched the points we made, sufficient for us to know whether he agrees or disagrees.

He says, "*Gain, profit or remuneration*, call it what we may, is, I am satisfied, the chief incentive to discoveries." It is true that the mass of so called inventors are very much influenced by the hope of gain, but the great majority of those who are influenced by gain never make discoveries of any real value. It is patent to every one who is at all familiar with such things, that almost every discovery and invention of any importance or real value, has been made independent of any such influence; they are usually made by persons possessing a native talent for invention and discovery.

The thousands of useless inventions are very striking and characteristic exhibitions of those who invent for gain.

Our correspondent does not pretend to gainsay the assertion that the dentist who advocates patents for any thing pertaining to his profession, arrays himself against the opinion and judgment of the best and most intelligent general practitioners of medicine of this and other countries, and at once forfeits their regard for, and appreciation of him, as a professional man.

The lost arts argument does not amount to any thing. The people in the days of old were not in a condition to appreciate and use inventions as now.

Any thing that was useful and valuable to mankind generally, was not and could not be lost.

Is invention and discovery a sufficiently sure method of making money to warrant any one to embark in it for that purpose. The tilt made at the "lauded *medical* view" of this subject is wholly gratuitous. The idea is held out here, that as the extent of the dentist's practice will warrant, up goes his prices from one dollar, step by step, to five or even ten dollars, as the case may be, for a single filling. He farther remarks, "In conversation with a dental acquaintance, on the subject of professional charges, the question was asked, What is the difference ordinarily between a three and a five dollar filling? The answer was, 'precisely two dollars.'" Now if he intended to say that this is all the difference, we take issue squarely, and hesitate not to say, that those who charge the largest fees make the best fillings; and that as an operator increases his fees he improves the character of his operations in a corresponding degree. It may be different in the acquaintance of our correspondent, but what we have stated is certainly true in the circle of our own acquaintance. This is the only true basis upon which to make an increase of fees.

We know of many operators who now charge from three to ten dollars for filling, and make less money than when they charged but one dollar. T.

### FREE NATION.

A paper bearing this title has recently been started in this city, under the editorial charge of Dr. C. B. Boynton, and Prof. H. V. N. Boynton.

It takes the position that religion should exercise a controlling influence over men in all circumstances, conditions and occupations; that the Bible should be a rule by which to measure *all* our acts, not only those which are religious, but also those of a social, civil, and political character.

We are pleased to see that this subject has been made a prominent point by this paper. Men everywhere are prone to forget that there is a supreme ruler who takes cognizance of all the minutiæ of their lives.

This paper labors to bring them back to a remembrance of this truth—and a great and glorious work it is.

We hope the paper will be well sustained, and not only sustained, but read, and that the editors will be encouraged to prosecute the good work in which they are engaged. T.

NAPKIN HOLDER.

We have used this instrument described on p. 225 of the present number of the Register, and find it to be very good in many cases.

By placing under it a roll of paper or cloth, the lips and cheeks are held away from the teeth nicely. For the lower teeth by using it in connection with Hawes' tongue-holder, the operator can employ his left hand for some other purposes than holding the mouth open, and the lips away from the point of work. Some operators have a great aversion to new things, at least till others have thoroughly tested them. This arises from a dislike to go out of the beaten path, or from parsimony. Every thing unless palpably worthless, that is presented to the profession, should receive consideration and examination, and if it stands the test, then it should be tried, and not only by a few, but by many, by all. Frequently, an instrument or appliance will be found valuable in the hands of some, while others will fail to appreciate and consequently to use it. The only way in which an operator can know whether a new instrument will be valuable in his hands, is to try it. Some boast of doing every thing with the smallest number of instruments; while the truly skillful and scientific operator will use such a variety as will meet most perfectly the exigencies of every case. It is not long since the old fashioned speculum was the only appliance of this kind, there are now eight or ten different instruments of this class, and all valuable—evidence of progress. T.

### SENSITIVE DENTINE.

We recently operated upon the teeth of Mrs. ——, which exhibited a peculiarity with regard to sensitiveness that we had not before observed to the same extent.

Ten teeth were filled ; in almost every instance upon opening the cavity and beginning to excavate, there was very little or no sensitiveness apparent ; but as the excavation proceeded, the tooth would become exceedingly sensitive in a few moments. This condition seemed to occur at the time to several of them ; chloride of zinc, creosote and iodine, was repeatedly applied without any lessening of the sensitiveness, the teeth were filled while in this condition, and the sensitiveness subsided after a short time.

The peculiarities of this case, viz.: the rapid occurrence of sensitiveness under the excavator, and the resistance manifested to the ordinary remedial agents, were such as we have not observed in any former case, and especially where there is not some well defined, predisposing cause, which there was not in this case. There was, however, rather less than the wonted strength and some irritability of the nervous system. Have others found cases of this kind, and what is the best course to pursue? T.

### LOST, STRAYED OR STOLEN.

The Dental Register of the West copies an editorial article from this Journal without credit.—*Medical & Surgical Reporter.*

The above we find in the Medical & Surgical Reporter, and we freely acknowledge the fact, that one of its editorials appeared amongst our *selections*, without credit. This was simply an oversight, and nothing more. The article is amongst our selections, and of course no one would think that we intended to appropriate it as our own in any respect, and it would strike every one as a simple omission to credit. We would just as soon give the Reporter credit for selections as any journal in the world.

We have no "*pick*" at the Reporter, indeed we think it a very nice journal, and a very good one, too, and we rather admire its spunky editor, and we like to see a man stand up for his rights, especially where a great principle is involved. Please forgive us, and we'll credit you a good deal next time.

A fellow at our elbow says, that it is difficult to tell which should have credit for the article in question, the Reporter or the London Lancet. But he's green. T

### FORCEPS.

The accompanying cut represents a forceps invented by Dr. M'Clelland, of Louisville, Ky.

The object of the instrument is to enable the operator to remove the second or third inferior molar, when the parts are swollen, and the mouth closed so that the ordinary forceps can not be introduced. The beaks are long and curved, so as to admit the crown of the anterior adjoining tooth between them, without being embraced; the points reaching on the tooth to be extracted and embracing it.

By this arrangement if the anterior teeth can be separated to the extent of the thickness of the joint of the instrument, which is about one-half an inch, the instrument may be introduced, and the tooth extracted. It is a valuable instrument—one that fills a niche—and should have a place in every dentist's case. The price is the same as the ordinary molar forceps. T.

A PRACTICAL TREATISE ON PHTHISIS PULMONALIS: *Embracing its Pathology, Causes, Symptoms and Treatment.* By L. M. LAWSON, M. D.

This work we have from the Publishers, Rickey, Mallory & Co. The work is prepared with particular reference to this disease as it exists in America. The author remarks, "It is not presumed that consumption is different in this country from the same disease elsewhere; but at the same time it is evident that the influences of climate, domestic habits, races, and other modifying conditions, render a systematic account of the disease as met with here, highly important." A systematic and well arranged work upon this subject is certainly of great importance. Consumption has been more prevalent, and more fatal in the United States than any other disease. This results, we doubt not, to a very great extent, from the fact that there has been no settled, well defined and systematic prophylactic and remedial treatment, with the physicians generally. There has been a great diversity of opinion in regard to the nature and treatment of this disease; some medicating extensively, and others doing nothing, each course about alike fallacious, judging by the results. In the work under notice the author occupies the entire ground, and we think, very thoroughly and practically. The nature of the disease, all its conditions and modifying influences, are discussed with great clearness, though with brevity; and all the material truths and facts are presented in such a way as not to be misunderstood. The influence of climate and locality upon the disease is fully discussed, as also the rationale of their operation.

The views and opinions of the author are entitled to very great regard, from the fact that he has made the study of this subject a life business, his practice in this disease has been very extensive, and whenever he presents an opinion of his own it is based upon personal observation.

The work is written in a very attractive style, and would be very attractive and interesting to the non-professional man, as well as the physician. No physician can in justice to himself and his patients be without this work. T.

## Figures and Tables

**Figure f1:**